# Does weight-for-height and mid upper-arm circumference diagnose the same children as wasted? An analysis using survey data from 2017 to 2019 in Mozambique

**DOI:** 10.1186/s13690-020-00462-7

**Published:** 2020-10-07

**Authors:** Tomás Zaba, Mara Nyawo, Jose Luis Álvarez Morán

**Affiliations:** 1 United Nations Children’s Fund, 1440 Zimbabwe Avenue, Maputo, Mozambique; 2 United Nations Children’s Fund, Eastern and Southern Africa Regional Office, Box 44145-00100, Nairobi, PO Kenya; 3 Independent Consultant, 2 Artichoke Hill, London, UK

**Keywords:** Wasting, WHZ, MUAC, Combined prevalence, Nutrition programme planning, Malnutrition

## Abstract

**Background:**

Three different diagnostic criteria are used to identify children aged 6 to 59 months with acute malnutrition: weight-for-height (WHZ), middle upper arm circumference (MUAC) and bilateral pitting oedema. Prevalence of malnutrition from surveys is among the most-used decision support data, however not all diagnostic criteria are used to calculate need, creating a mismatch between programme planning and implementation. With this paper, we investigate if such discrepancies are observed in Mozambique.

**Methods:**

Population-based nutritional anthropometric surveys from 45 districts in Mozambique conducted by the Technical Secretariat for Food Security and Nutrition (SETSAN) and UNICEF between 2017 and 2019 were analysed. We used Cohen’s kappa coefficient to measure inter-rater agreement between WHZ and MUAC, Spearman’s rank-order coefficient to assess the correlation, binary logistic regression to investigate factors influencing WHZ and MUAC diagnostic classification. We compared acute malnutrition caseload estimates by WHZ, MUAC and oedema to caseloads from combined prevalence estimates.

**Results:**

WHZ and MUAC rarely agree on their diagnostic classification (κ = 0.353, ρ < 0.001) and results did not vary by province. We found positive correlation between WHZ and MUAC (rho = 0.593, *ρ* < 0.0001). Binary logistic regression explained 3.1% of variation in WHZ and 12.3% in the MUAC model. Girls (AOR = 1.6, *ρ* < 0.0001), children < 24 months (AOR = 5.3, *ρ* < 0.0001) and stunted children (AOR = 3.5, *ρ* < 0.0001) influenced the MUAC classification. In the WHZ model, children < 24 months (AOR = 2.4, *ρ* < 0.0001) and stunted children (AOR = 1.7, *ρ* < 0.0001) influenced the classification, sex had no effect. Caseload calculations of global acute malnutrition by WHZ and/oedema-only and by MUAC and/oedema-only yielded less children than caseload calculations using the combined prevalence estimates. Similarly, caseload calculations for SAM by WHZ and/oedema-only and SAM by MUAC and/oedema-only yielded less children than the respective combined prevalence calculations.

**Conclusions:**

Given the discrepancy in diagnostic classification between WHZ and MUAC in Mozambique, using either one alone for calculating burden underestimates the real number of children in need of treatment and negatively affects nutrition programme planning. We recommend that use of the combined prevalence estimates, based on the three diagnostic criteria of WHZ, MUAC and oedema, be officially adopted. Further analysis is needed to detail the programmatic impact of this change.

## Background

Acute malnutrition in children under 5 years is a life-threatening and devastating disease of epidemic proportions world-wide, and especially in low and middle-income countries [1]. Wasting, or acute malnutrition, is diagnosed using weight-for-height z-scores (WHZ) or mid-upper arm circumference (MUAC) [2]. Its prevalence is estimated through representative population-based surveys which are carried out at national and/or sub-national level as needed. Based on their findings, response plans are put in place to prevent further deterioration of the nutrition situation [3]. The clinical sign of bilateral pitting oedema is another criteria used to diagnose acute malnutrition [4]. This paper will focus on the two anthropometric indicators.

Practical experience as well as research from different countries has shown that WHZ and MUAC identify different children as being wasted, with a small overlap between the two measurers as described below. These discrepancies have an implication when measuring the prevalence of acute malnutrition and classifying the severity of an area which guides decisions around the need to set up an intervention. This issue was raised during Integrated Phase Classification for Acute Malnutrition (IPC for AMN) workshops in Mozambique, where it was noted that in some areas the prevalence of Global Acute Malnutrition (GAM) by MUAC was higher than GAM by WHZ [5, 6].

Evidence from the literature has consistently found that MUAC and WHZ are discrepant. A study conducted by Laillou et al., (2014) in Cambodian children using a secondary data analysis of 11,000 datasets from 2010 and 2012, found that the prevalence of wasting was 3.3% using MUAC compared to 10.6% when WHZ was used [7]. Contrary to this, a study conducted in southern Ethiopia by Tadesse et al., (2017, p.5) found that “MUAC categorized more children as wasted (10.5%, 95% CI: 9,6%- 11,4%) compared to WHZ (5.4%, 95% CI: 4.8%-6.1%)” [8].

Still in Cambodia, Wieringa et al., (2018) with the aim of exploring factors associated with wasting by MUAC and by WHZ and using longitudinal data of 4381 children, found that WHZ continued to identify higher rates of wasting than MUAC (14.4 and 10.1% respectively). Looking at associations in a multinomial regression model, factors associated with wasting diagnosed by WHZ included being older (*p* < 0,01), being stunted and being male (males were 1.9 times more likely to be wasted); while for MUAC the associated factors were being younger, being female (females were 3.2 times more likely to be wasted) and being stunted (stunted children were 4.9 times more likely to be wasted) and the differences were all statistically significant (*p* < 0,05) [7]. It should be noted that stunting prevalence was 19.7%, which is much lower than stunting prevalence in Mozambique, which is 43% [9].

A recently published paper by Bilukha and Leidman (2018) analysed 773 population-representative anthropometric surveys from 41 countries that were conducted by *Action Contre la Faim* (ACF) and the United Nations High Commission for Refugees (UNHCR) between 2001 and 2016. They found that the median prevalence of wasting by WHZ was 10.47%, while by MUAC wasting was 6.66% (Bilukha and Leidman, 2018 p.1). Mozambique was one of the 41 countries included in the analysis (contributing one survey with 406 children) and the prevalence of wasting by WHZ and MUAC showed the same value of 3.26% with no confidence interval available. Another study by Grellety & Golden (2016), analysed data from 47 countries in order to compare the degree and direction of discrepancy between WHZ and MUAC across countries. Again, Mozambique was included, contributing 14 surveys with an overall 3828 children aged 6 to 59 months. Results for Mozambique showed that of the total cases of acute malnutrition (579), 21% where identified by WHZ < -2 while over 43.9% were identified by MUAC < 125 mm [8].

In Mozambique, although most surveys collect MUAC as well as weight and height measurements, acute malnutrition prevalence estimates use WHZ and/or oedema only. The Mozambique National Protocol for treatment of children with acute malnutrition follows WHO recommendations whereby programme admissions are made using either WHZ or MUAC classifications, as well as oedema [9–11]. However, for programme planning, estimates of the number of children in need of treatment are calculated using prevalence obtained by WHZ only. Despite a well-documented recognition that children with a low MUAC have an increased risk of death [12], WHZ has traditionally been preferred over MUAC when estimating prevalence, and MUAC-based prevalence estimates have only been used in the absence of WHZ. This situation has changed somewhat with the recent updates made in the new IPC Acute Malnutrition Version 3 guidelines. MUAC-based prevalence can now be used to classify severity even when WHZ-based prevalence estimates also exist, as long as there is historical data showing the relationship between MUAC and WHZ in the context under analysis [13].

The objective of the present study is to understand the following research questions: Is there any agreement between WHZ and MUAC in Mozambique when identifying wasting? How do WHZ and MUAC correlate in Mozambique and what are the possible factors that explain discrepancies? How does use of the combined estimates change the prevalence and burden estimates in Mozambique? How different is the combined prevalence using WHZ and MUAC and/oedema from either WHZ and/oedema-only or MUAC and/oedema-only prevalence? Considering that these questions have not previously been analysed in Mozambique, we believe that analysing existing data to answer these questions will improve programme planning and implementation of the national protocol for treatment of acute malnutrition.

## Methods

### Source of data

The study used 46 district level surveys that were implemented in 45 different districts (one district had 2 surveys, of total 161 districts in Mozambique) between 2017 and 2019. Almost all of the 11 Provinces of Mozambique were represented in the study sample, except for Niassa and Maputo Cidade. All surveys were population-based, and designed to be representative at district level following the SMART methodology [14]. The surveys were conducted in order to assess the need for emergency nutrition programmes and were led by the Government through the Technical Secretariat for Food Security and Nutrition (SETSAN) and Partners. All surveys used a two-stage cluster sampling approach with probability proportional to size of the population applied in stage 1, following the SMART methodology. Anthropometric measurements were taken using the same instruments across all surveys and enumerators were trained and submitted to a standardization test. A calendar of local events was used in all surveys to estimate age in months for children without official document to extract birthdate.

### Data processing

Data was initially cleaned using ENA for SMART software version July 9th, 2015 [14] and Z-scores were calculated according to the WHO 2006 growth reference. SMART flags (±3 z-scores) were used to exclude non plausible WHZ data and overall quality of data was verified through the SMART plausibility check in ENA to ensure that all data used met documented quality standards [14]. There were no exclusion criteria for MUAC. Datasets were then imported to IBM SPSS version 25 (IBM Corp. Released, 2016) where further analysis was carried out. Wasting by WHZ was defined as <− 2 Z-scores and wasting by MUAC as < 125 mm.

The first objective of the analysis was to ascertain if WHZ and MUAC classified the same children as wasted. Being a binary test, which can return only two possible values, Cohen’s Kappa (k) coefficient was used to measure the inter-rater agreement between diagnosis of wasting by WHZ and by MUAC [15–17] and results were split by province. The second objective was to see how the two indicators (WHZ and MUAC) correlated. Since MUAC distribution was not normal as per the Kolmogorov-Smirov test of normality (D = 0.0225, *p* < 0.0001), a Spearman’s rank-order correlation was run [18, 19]. The third objective was to identify what factors are associated with the observed discrepancy between WHZ and MUAC and to calculate the corresponding odds ratios. To do so, a binary logistic regression applying the enter method was used [20, 21] with first wasting by WHZ as the dichotomous dependent variable, and sex (boys as reference), age in two categories (≥24 months as reference), and presence of stunting (“no” as reference) as independent variables; secondly, wasting by WHZ was replaced with wasting by MUAC as the dependent variable and the independent variables remained the same. For the analysis of difference between WHZ and MUAC bilateral pitting oedema was excluded.

**Fig. 1:**
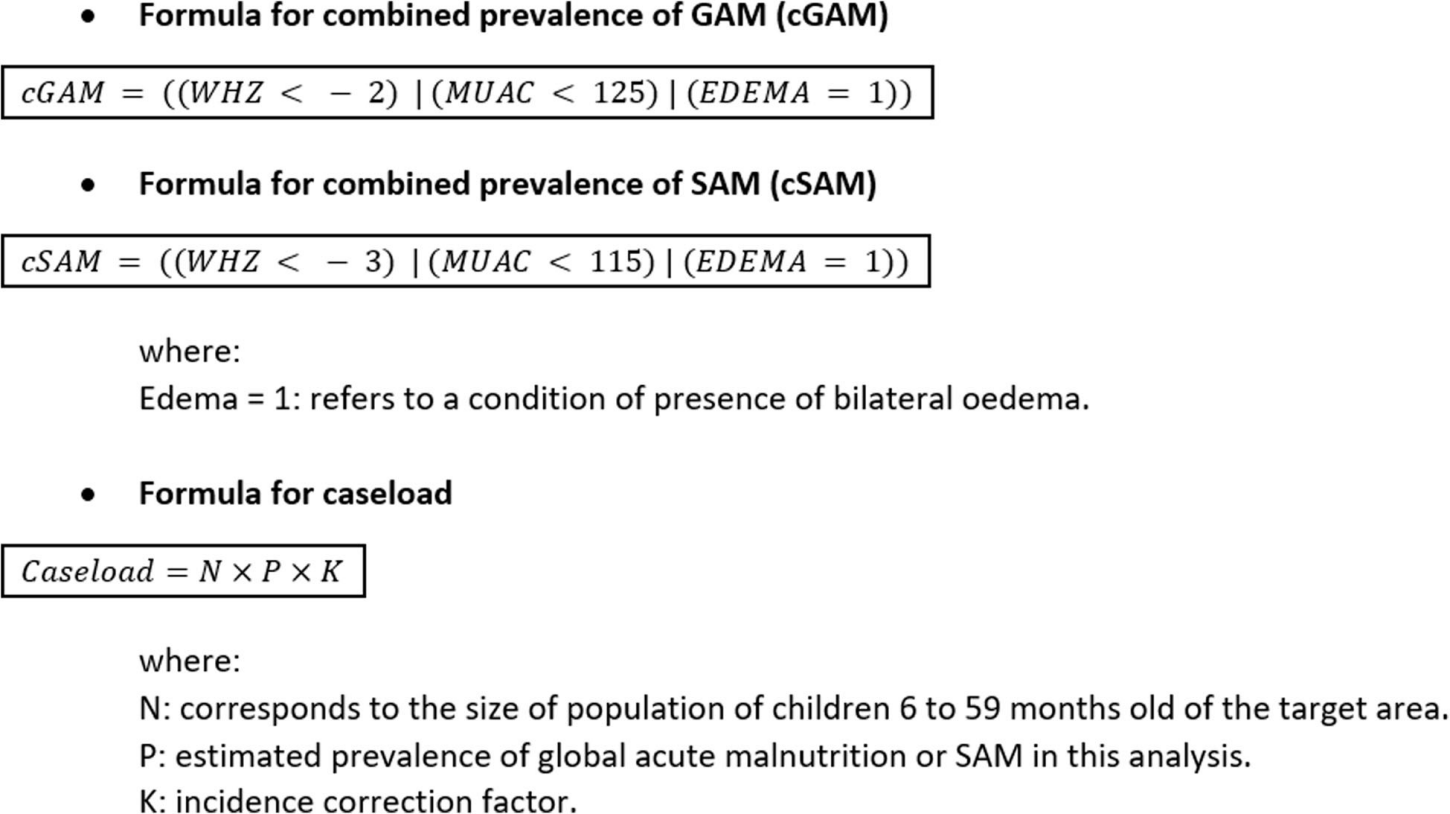
Description of the formulas used for calculating the combined prevalence and caseload

For the analysis of combined prevalence, bilateral pitting oedema was included, and was then called Global Acute Malnutrition (GAM). GAM is clinically divided into severe acute malnutrition (SAM) and moderate acute malnutrition (MAM) [2]. Combined GAM (cGAM) included children with WHZ < -2 and/or MUAC < 125 and/or presence of bilateral pitting oedema, and combined prevalence of SAM (cSAM) included children with WHZ < − 3 and/or MUAC < 115 and/or presence of bilateral pitting oedema. Caseload was then calculated by applying the formula as described in Fig. 1 and using an incidence correction factor of 2.6 [22].

## Results

There were 12,639 children aged between 6 and 59 months included in our analysis, from the 46 surveys in 45 districts distributed across 9 (of 11) provinces in Mozambique. Mean age was 30.4 ± 14.8 months, 37.9% of the children were less than 24 months and 50.2% were girls. Mean MUAC was 149 mm ± 13 mm with ranges between 93 mm and 211 mm. MUAC flags were not used. Mean WHZ was − 0.0045 ± 1.03 with ranges between − 3.589 z-scores and 3.310 z-scores. Flags for extreme values for WHZ were removed.

Overall, prevalence of wasting by MUAC was higher than WHZ in the majority of districts (58.7%). It was interesting to observe that unlike other provinces included in this analysis, in Zambézia wasting by MUAC was higher in 8 of the 9 districts included in the study and in Cabo Delgado wasting by MUAC was higher in 4 of the 5 districts included in the study. Further details are presented below in Table 1.

**Table 1:** Prevalence of wasting by WHZ only and by MUAC only in 45 districts surveyed between 2017 and 2019 in Mozambique

Province	District	Number of children	Prevalence of wasting by WHZ, 95% CI	Prevalence of wasting by MUAC, 95% CI
Cabo Delgado	Balama	237	3.4% (1.06–5.7)	7.6%, (4.2–10.9)
	Ibo	256	10.9% (7.1–14.8)	7%, (3.8–10.1)
	Mecufi	323	4.6% (2.3–7.0)	8.7%, (5.6–11.8)
	Meluco	259	3.5% (1.2–5.7)	4.2%, (1.7–6.7)
	Namuno	363	5.2% (2.9–7.5)	6.9%, (4.3–9.5)
Nampula	Mogovolas	378	4.2% (2.2–6.3)	4%, (2.0–6.0)
	Monapo	353	2% (0.9–4.2)	5.9%, (3.5–8.4)
	Nacala-a-Velha	270	7% (4.0–12.4)	7.8%, (4.6–10.9)
	Ribaue	380	2.6% (1.4–5.1)	2.1%, (0.6–3.6)
Zambézia	Maganja da Costa	197	2% (0.7–5.5)	5.1%, (2.0–8.2)
	Milange	338	3.8% (1.8–5.9)	6.5%, (3.9–9.2)
	Morrumbala	354	4.5% (3.5–6.7)	6.8%, (4.1–9.4)
	Namacurra	207	2.9% (1.2–7.0)	3.9%, (1.2–6.5)
	Nicoadala	262	0.8% (0.2–3.0)	5.7%, (2.8–8.5)
	Gurue	336	2.4% (1.1–5.3)	4.5%, (2.2–6.7)
	Lugela	321	4% (2.4–7.0)	5.3%, (2.8–7.6)
	Molumbo	288	4.2% (2.4–7.3)	4.9%, (2.3–7.3)
	Pebane	407	4.2% (2.5–7.0)	4.2%, (2.3–6.1)
Tete	Cahora-Bassa	341	2.6% (0.9–4.4)	1.8%, (0.4–3.2)
	Changara	235	2.1% (0.2–4.0)	2.1%, (0.2–4.0)
	Doa	222	5.4% (2.4–8.5)	9%, (5.2–12.8)
	Moatize	198	1% (− 0.4–2.4)	2.5%, (0.3–4.7)
	Mutarara (in 2019)	214	3.7% (1.2–6.3)	4.7%, (1.8–7.5)
	Mutarara (in 2018)	395	2.3% (0.8–3.4)	3%, (1.3–4.7)
	Chiuta	218	5% (2.1–8.0)	1.8%, (0.04–3.6)
	Magoe	212	4.2%, (1.5–7.0)	1.9%, (0.04–3.7)
Sofala	Beira	224	4.5%, (2.3–8.7)	2.7%, (0.5–4.8)
	Buzi	187	1.6%, (0.6–4.6)	2.7%, (0.3–5.0)
	Caia	230	3.5% (1.6–8.0)	2.2%, (0.3–4.1)
	Dondo	178	4.5%, (2.1–10.1)	1.1%, (− 0.4–2.7)
	Nhamatanda	205	2.9%, (1.3–7.0)	1%, (− 0.4–2.3)
Manica	Gondola	297	2.7%, (0.8–4.5)	1.3%, (0.03–2.7)
	Macossa	237	2.5%, (0.5–4.5)	5.1%, (2.3–7.9)
	Sussundenga	186	1.6%, (− 0.2–3.4)	1.6%, (− 0.2–3.4)
	Tambara	197	2%, (0.04–4.0)	6.1%, (2.7–9.5)
Inhambane	Funhalouro	245	1.2%, (− 0.16–2.6)	0.4%, (− 0.4–1.2)
	Govuro	281	1.1%, (− 0.14–2.3)	1.4%, (0.03–2.8)
	Panda	253	0%, (0.0–0.0)	1.6%, (0.03–3.1)
Gaza	Chibuto	277	1.8%, (0.2–3.4)	0.4%, (− 0.4–1.1)
	Chicualacuala	273	0.4%, (− 0.4–1.1)	1.5%, (0.03–2.9)
	Chigubo	282	1.1%, (− 0.1–2.3)	1.4%, (0.03–2.8)
	Guija	279	1.4%, (0.03–2.8)	1.8%, (0.2–3.4)
	Mabalane	374	0.3%, (− 0.3–0.8)	0.8%, (− 0.1–1.7)
Maputo Provincie	Magude	265	1.5%, (0.03–2.9)	1.1%, (− 0.1–2.4)
	Manhica	266	4.1%, (1.7–6.5)	3%, (0.9–5.1)
	Namaacha	339	2.7%, (0.9–4.4)	2.1%, (0.5–3.6)
TOTAL		12,639		

### Level of agreement between WHZ and MUAC in diagnosing wasting

**Fig. 2:**
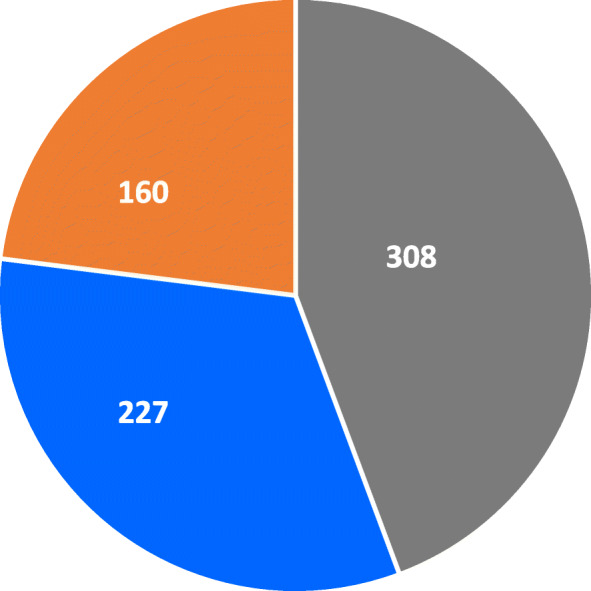
Pie chart showing number of wasting cases diagnosed using MUAC < 125 mm only (grey colour), WHZ < -2 Z-score only (blue colour) and the overlap (orange colour) for the pooled datasets of nutrition surveys conducted in 45 districts between 2017 and 2019 in Mozambique

Results from the Cohen’s κ test show a minimal agreement between the two diagnostic tests, k = 0.353, *ρ* = 0.000, meaning that overall between 4 to 15% of data points for each child agree on the diagnosis of wasting by WHZ and by MUAC (Fig. 2). At provincial level the same pattern was observed with some highlights in Sofala and Inhambane provinces where a very low level of agreement was observed, and only 0–4% of data agreed on the diagnosis of wasting by WHZ and by MUAC. A weak level of agreement was observed in Maputo province, corresponding to 15–35% agreement (Table 2). Figure 2 illustrates the larger number of children identified as wasted using MUAC and shows the level of overlap between the two diagnostic criteria.

**Table 2:** Results of the Cohen’s Kappa test for the pooled datasets grouped by Province from nutrition surveys conducted from 2017 to 2019 in Mozambique

	Value of Kappa	Level of agreement^a,b^	*ρ*-value
Pooled dataset	0.353	Minimal	< 0.001
Results split by province			
Cabo Delgado	0.363	Minimal	< 0.001
Nampula	0.385	Minimal	< 0.001
Zambézia	0.387	Minimal	< 0.001
Tete	0.377	Minimal	< 0.001
Sofala	0.086	None	0.004
Manica	0.288	Minimal	< 0.001
Inhambane	0.125	None	< 0.001
Gaza	0.25	Minimal	< 0.001
Maputo province	0.464	Weak	< 0.001

^a^Level of agreement taken from McHugh (2012)

^b^Level of agreement according to value of Kappa: None: 0–0.20; Minimal: 0.21–0.39; Weak: 0.40–0.59; Moderate: 0.60–0.79; Strong: 0.80–0.90; Almost perfect: above 0.90.

### Correlation between WHZ and MUAC

**Fig. 3:**
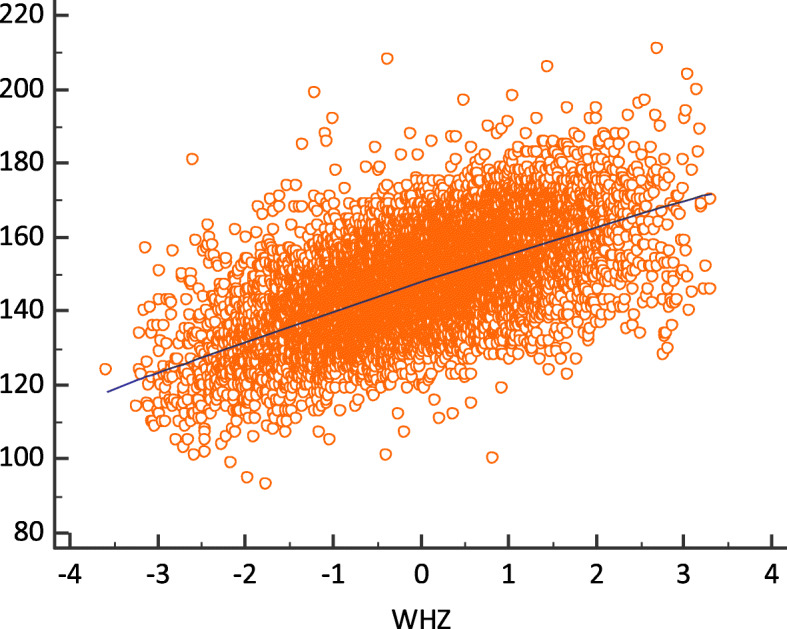
Scatter diagram of Spearman rank correlation with MUAC scores on Y axis and WHZ scores on X axis, from the pooled datasets of nutrition surveys conducted in 45 districts from 2017 to 2019 in Mozambique. Each orange dot corresponds to a point where MUAC scores and WHZ scores are linked. The blue line is the linear fit

There was a positive correlation between MUAC scores and WHZ scores, which was statistically significant (rho = 0.593, *ρ* < 0.0001). As MUAC score increases, so does WHZ score and vice-versa, suggesting that the observed discrepancies could be explained by other factors (Fig. 3).

### Factors associated with wasting by WHZ and wasting by MUAC

Two binary logistic regression tests were performed to ascertain the effects of age, sex and stunting on the diagnosis of wasting using WHZ and using MUAC. The two tables below show the results of the regression model for wasting by WHZ (Table 3) and the regression model for wasting by MUAC (Table 4). Overall, the model presented in Table 3 was able to explain only 3.1% of the variation in WHZ diagnosis and the model was statistically significant (X^2^ = 94.071, *ρ* < 0.0001), while the model presented in Table 4 explained 12.3% of the variation in MUAC diagnosis, and was also statistically significant (X^2^ = 430.429, *ρ* < 0.0001). This means that there are many other factors that contribute to the variation seen, in addition to the factors included in each model.

**Table 3:** Binary logistic regression model for wasting by WHZ. Results are presented as the overall of the 45 districts and breakdown by province, using the pooled dataset of nutrition surveys collected between 2017 and 2019 in Mozambique

Step 1		B	Sig.	Exp(B)	95% C.I. for Exp(B)	
					Lower	Upper
Overall	Sex (girls)	−0.168	0.108	0.846	0.689	1.037
	Age (< 24 months)	0.865	**0.000**	2.375	1.934	2.918
	Stunting (yes)	0.523	**0.000**	1.686	1.375	2.068
Cabo Delgado	Sex (girls)	0.087	0.711	1.091	0.688	1.73
	Age (< 24 months)	1.132	**0.000**	3.102	1.935	4.972
	Stunting (yes)	0.251	0.286	1.285	0.811	2.038
Nampula	Sex (girls)	0.124	0.663	1.132	0.647	1.981
	Age (< 24 months)	0.549	0.054	1.731	0.99	3.027
	Stunting (yes)	0.341	0.237	1.407	0.799	2.476
Zambézia	Sex (girls)	−0.511	**0.022**	0.6	0.388	0.928
	Age (< 24 months)	0.992	**0.000**	2.696	1.74	4.176
	Stunting (yes)	0.374	0.084	1.453	0.951	2.219
Tete	Sex (girls)	0.074	0.77	1.077	0.655	1.772
	Age (< 24 months)	0.629	**0.013**	1.875	1.14	3.084
	Stunting (yes)	1.041	**0.000**	2.832	1.688	4.754
Sofala	Sex (girls)	−0.946	**0.013**	0.388	0.184	0.82
	Age (< 24 months)	1.053	**0.003**	2.866	1.422	5.776
	Stunting (yes)	0.194	0.598	1.214	0.591	2.492
Manica	Sex (girls)	−0.938	0.056	0.392	0.15	1.022
	Age (< 24 months)	0.771	0.084	2.161	0.903	5.172
	Stunting (yes)	0.326	0.469	1.386	0.573	3.35
Inhambane	Sex (girls)	16.948	0.993	> > 100^a^	n.a^b^	n.a^b^
	Age (< 24 months)	1.248	0.153	3.483	0.629	19.279
	Stunting (yes)	−0.485	0.661	0.615	0.071	5.368
Gaza	Sex (girls)	0.211	0.698	1.235	0.426	3.584
	Age (< 24 months)	0.723	0.183	2.061	0.71	5.983
	Stunting (yes)	−1.263	0.225	0.283	0.037	2.176
Maputo province	Sex (girls)	−0.213	0.613	0.808	0.354	1.845
	Age (< 24 months)	0.508	0.223	1.662	0.735	3.761
	Stunting (yes)	0.49	0.309	1.632	0.635	4.198

^a^This province had only 6 cases of wasting and all of them were girls. ^b^ not applicable

**Table 4:** Binary logistic regression model for wasting by MUAC. Results are presented as the overall of the 45 districts and breakdown by province using the pooled dataset of nutrition surveys collected between 2017 and 2019 in Mozambique

Step 1		B	Sig.	Exp(B)	95% C.I. for Exp(B)	
					Lower	Upper
Overall	Sex (girls)	0.473	**0.000**	1.605	1.325	1.945
	Age (< 24 months)	1.668	**0.000**	5.304	4.297	6.547
	Stunting (yes)	1.244	**0.000**	3.47	2.852	4.221
Cabo Delgado	Sex (girls)	0.809	**0.000**	2.247	1.433	3.522
	Age (< 24 months)	2.062	**0.000**	7.864	4.779	12.939
	Stunting (yes)	1.211	**0.000**	3.357	2.113	5.333
Nampula	Sex (girls)	0.519	**0.049**	1.68	1.002	2.818
	Age (< 24 months)	1.48	**0.000**	4.393	2.527	7.638
	Stunting (yes)	0.843	**0.002**	2.323	1.359	3.972
Zambézia	Sex (girls)	0.15	0.394	1.162	0.822	1.643
	Age (< 24 months)	1.739	**0.000**	5.693	3.808	8.511
	Stunting (yes)	0.758	**0.000**	2.135	1.504	3.031
Tete	Sex (girls)	0.621	**0.019**	1.86	1.108	3.122
	Age (< 24 months)	1.576	**0.000**	4.837	2.799	8.36
	Stunting (yes)	1.653	**0.000**	5.224	2.935	9.298
Sofala	Sex (girls)	0.772	0.109	2.164	0.842	5.563
	Age (< 24 months)	1.283	**0.008**	3.607	1.407	9.251
	Stunting (yes)	1.859	**0.000**	6.419	2.416	17.057
Manica	Sex (girls)	0.69	0.076	1.993	0.931	4.267
	Age (< 24 months)	1.665	**0.000**	5.286	2.388	11.699
	Stunting (yes)	1	**0.012**	2.718	1.247	5.921
Inhambane	Sex (girls)	0.116	0.866	1.123	0.294	4.293
	Age (< 24 months)	0.786	0.25	2.195	0.575	8.382
	Stunting (yes)	2.496	**0.002**	12.139	2.493	59.095
Gaza	Sex (girls)	1.542	**0.016**	4.675	1.332	16.414
	Age (< 24 months)	1.427	**0.008**	4.165	1.447	11.992
	Stunting (yes)	0.843	0.104	2.324	0.84	6.43
Maputo province	Sex (girls)	−0.472	0.357	0.624	0.228	1.705
	Age (< 24 months)	1.153	**0.02**	3.168	1.203	8.341
	Stunting (yes)	1.393	**0.004**	4.027	1.541	10.525

Age and stunting were the variables with a statistically significant difference (*ρ* < 0.001) in the model by WHZ. The model predicted that younger children (less than 24 months) were 2.4 times more likely to be diagnosed as wasted using WHZ than older children. Stunted children were 1.7 times more likely to be diagnosed as wasted using WHZ than non-stunted children. Being either a boy or a girl had no effect on the diagnosis of wasting by WHZ. Breakdown analysis by province showed an increased likelihood to be diagnosed positive for wasting using WHZ in Cabo Delgado (3.1 times more likely), Zambézia (2.7 times more likely), Tete (1.9 times more likely) and Sofala (2.9 times more likely).

Table 4 shows results of the regression model for wasting by MUAC. As per this model, MUAC-based diagnosis is significantly influenced by all three factors considered with increased odds ratios for sex, age and stunting (*ρ* < 0.001). Girls were 1.6 times more likely to be diagnosed as wasted when using MUAC, children aged less than 24 months were 5.3 times more likely to be wasted than older children, and stunted children are 3.5 times more likely to be wasted than non-stunted children. Breakdown analysis by province is presented below in Table 4.

### Programmatic implications of the difference between WHZ-only prevalence and MUAC-only prevalence

These findings clearly highlight the fact that WHZ and MUAC diagnose different children as wasted and that they rarely agree, meaning that they rarely identify the same children. From a programmatic point of view, this difference results in many children who are not included in programme planning – including for advocacy, supply planning, human resource and financial needs – when WHZ-only or MUAC-only diagnostic criteria are used. As a result, the Nutrition Sector combines both WHZ and MUAC estimates (as proposed by Grellety & Golden, 2016) to ensure that all children who are acutely malnourished and in need of life-saving treatment are considered when planning programme responses and estimating numbers in need.

### Combined prevalence of global acute malnutrition

**Fig. 4:**
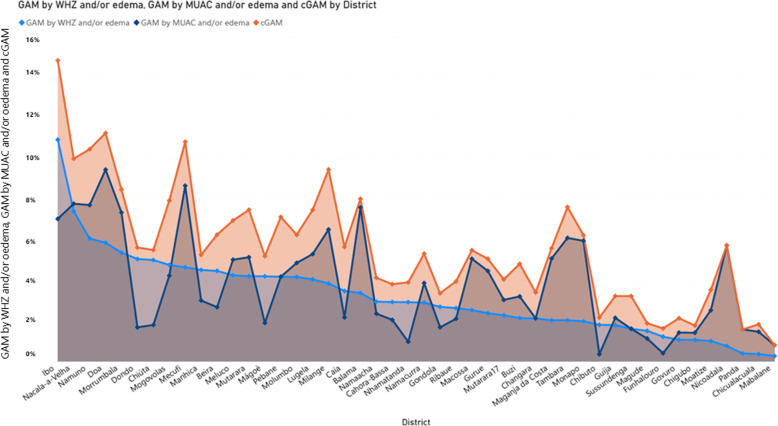
GAM by WHZ, GAM by MUAC and cGAM trade-offs across the 45 districts using the pooled dataset from nutrition surveys collected between 2017 and 2019 in Mozambique . The graph was plotted in Microsoft Power Business Intelligence sorted from the highest prevalence observed for GAM by WHZ and/or oedema

**Fig. 5:**
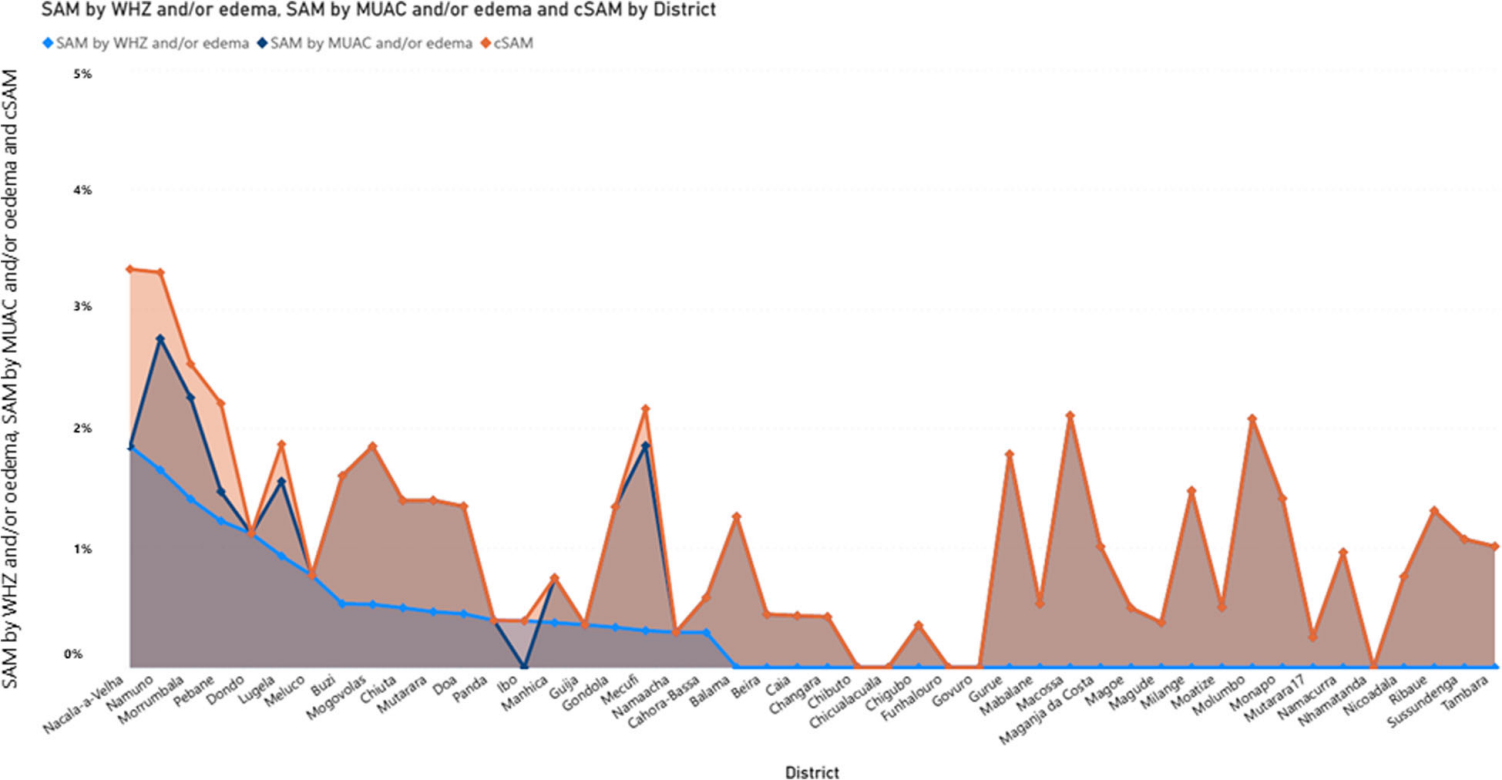
SAM by WHZ and/or oedema, SAM by MUAC and/or oedema and cSAM trade-offs across the 45 districts using the pooled dataset from nutrition surveys collected between 2017 and 2019 in Mozambique. The graph was plotted in Microsoft Power Business Intelligence sorted from the highest prevalence observed for SAM by WHZ and/or oedema

Since there were not many cases of bilateral pitting oedema observed, GAM by WHZ and GAM by MUAC presented in Fig. 4 follows the same distribution as reported in Table 1. Looking at cGAM, prevalence ranges from 0.80 to 14.84%. The same pattern was observed for SAM, where SAM by MUAC and/or oedema diagnosed more children than SAM by WHZ and/or oedema (Fig. 5) and for the combined prevalence (cSAM).

### Number of children in need of treatment using cGAM and cSAM

**Fig. 6:**
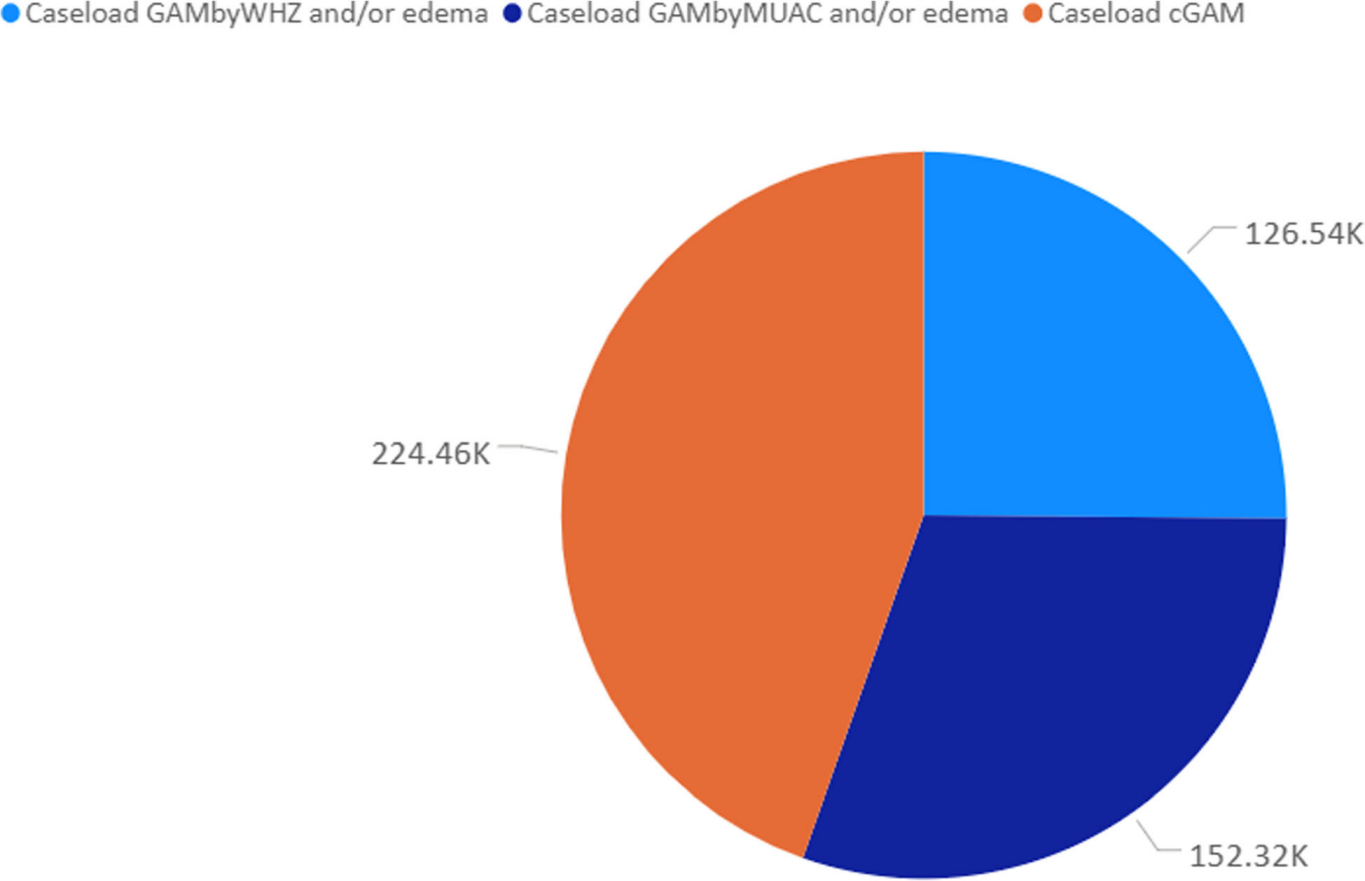
Total number of children (in thousands) in need of treatment using GAM by WHZ only, GAM by MUAC only and cGAM (combined GAM). The letter “K” after data value outside the pie means thousands, so that 126.54 K (rounded up to 127 K) is read as one hundred and twenty-seven thousand. Calculation made using census population 2017 and pooled dataset of nutrition surveys collected between 2017 and 2019 in Mozambique

**Fig. 7:**
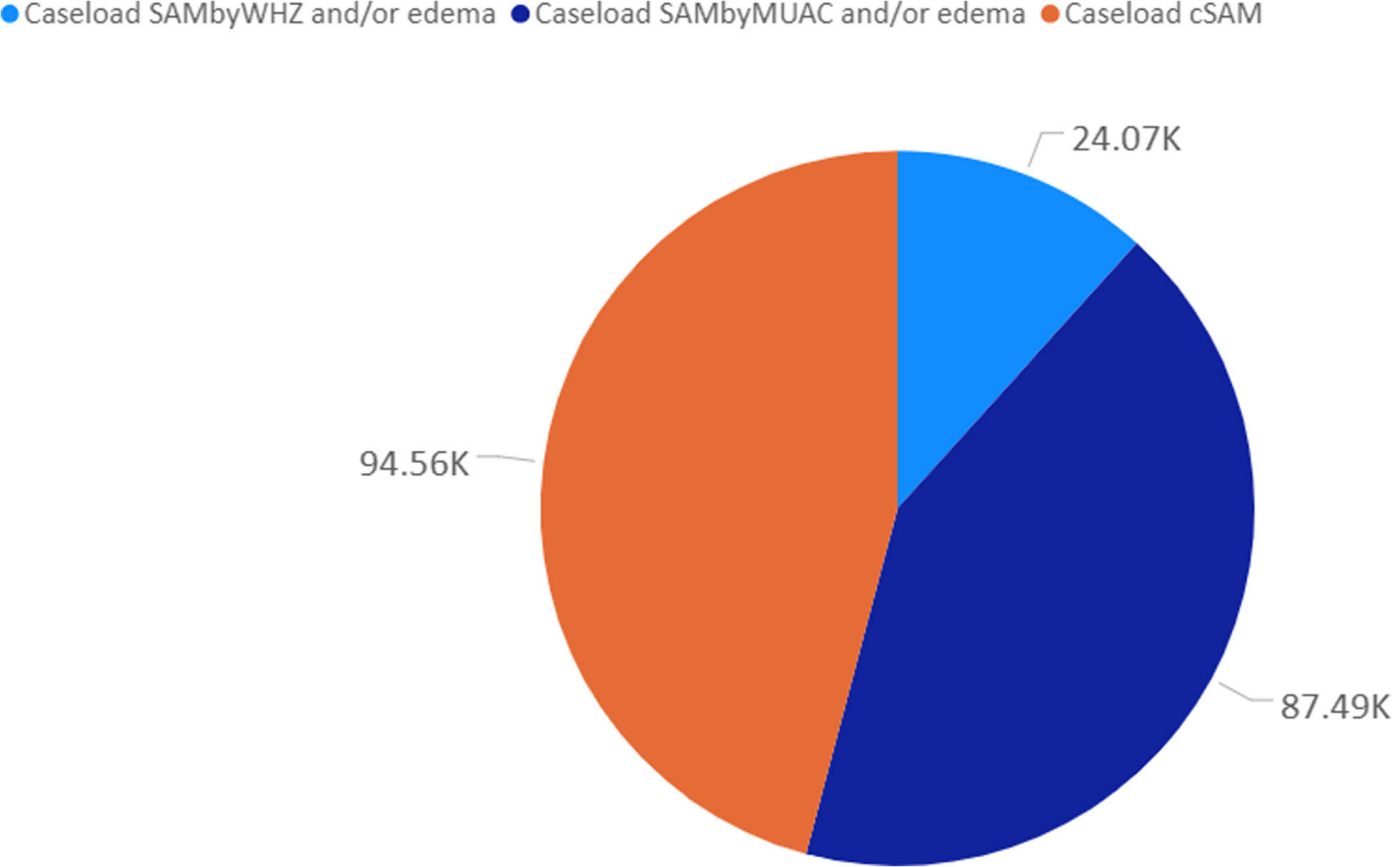
Total number of children (in thousands) in need of treatment using SAM by WHZ and/or oedema, SAM by MUAC and/or oedema and cSAM. The letter “K” after data value outside the pie means thousands, so that 24.07 K (rounded down to 24 K) is read as twenty-four thousand. Calculation made using census population 2017 and pooled dataset of nutrition surveys collected between 2017 and 2019 in Mozambique

The caseload calculation for a 12-month scenario considering an incidence correction factor of 2.6 shows some discrepancies when using cGAM, GAM by WHZ-only and GAM by MUAC-only as expected. Using cGAM gave an estimated 224,000 children expected to suffer from acute malnutrition during 1 year, while using GAM by WHZ and/oedema-only yielded 127,000 children corresponding to 43.3% less (Fig. 6). Using GAM by MUAC and/or oedema-only yielded 152,000 children, corresponding to 32.1% less than cGAM estimates. For SAM, the combined estimates (cSAM) yielded 95,000 children in need of treatment (Fig. 7), while using SAM by WHZ and/oedema-only yielded 24,000 (74.7% less) and SAM by MUAC and/oedema 87,000 children (8.4% less).

## Discussion

Our analysis sought to investigate whether there are discrepancies between WHZ and MUAC diagnostic criteria, to explore what may explain the discrepancies and what the potential implications may be for nutrition programme planning and design in Mozambique. We used a large sample from pooled surveys with representation of nine out of the eleven provinces in Mozambique.

Our study findings show that in Mozambique WHZ and MUAC classifications of wasting very rarely agree as assessed by Kohen’s test (Table 2). This is observed in all the provinces included in this analysis. These results are not surprising because they corroborate field level observations as well as results reported from studies in other countries. Studies across Africa and other regions have shown similar levels of discordance, showing WHZ diagnosing more children than MUAC [23] in some locations, while in other locations MUAC diagnoses more children than WHZ [8, 24]. Our analysis shows that in the case of Mozambique, MUAC diagnoses more children than WHZ (Fig. 2). Our findings are different to those reported by Bilukha & Leidman (2018) which showed no difference between WHZ and MUAC for Mozambique. A reason for this could be that our analysis comprises many more data points (12,639 compared to Bilukha and Leidman’s 406), and greater geographical representation across the country.

With regards to the correlation between WHZ and MUAC, our study findings show that when values for WHZ increase, so do values for MUAC, and vice-versa as per the Spearman’s correlation (rho) (Fig. 3). This suggests existence of other factors, in addition to those tested in this study, influencing the observed diagnostic discrepancy. Our findings are not distant from others. Bilukha & Leidman (2018), with data from almost all continents, found a positive correlation between WHZ and MUAC (above 0.5). The correlation coefficient was lowest (rho = 0.3553) in Eastern and Southern Africa.

When looking at factors that may explain the discrepancy between WHZ and MUAC diagnosis our binary logistic regression, using the same explanatory variables, was able to explain 3.1% of the variation in WHZ and 12.3% of the variation in MUAC with both models fitting significantly well (X^2^ = 94.071, *ρ* < 0.0001 for WHZ and X^2^ = 430.429, *ρ* < 0.0001 for MUAC). Consistent with evidence in the literature, in our models stunted children have a significantly increased likelihood of being diagnosed as wasted by MUAC (AOR = 3.5, *ρ* < 0.0001), younger children (less than 24 months) have a higher likelihood of being diagnosed as wasted by MUAC (AOR = 5.3, *ρ* < 0.0001) and females also have an increased likelihood of wasting (AOR = 1.6, *ρ* < 0.0001) [25]. Our findings of gender were different from Bilukha & Leidman’s (2018) results which found that the “proportion of females in the sample was not significantly associated with prevalence of wasting by MUAC”. In our study the significance of association between these three factors did not change by province, and further increased the AOR (Table 3 for WHZ and Table 4 for MUAC), especially in Cabo Delgado (3.1 times more likely), Zambezia (2.7 times more likely), Tete (1.9 times more likely) and Sofala (2.9 times more likely). Our analysis cannot explain these variations in our results between provinces, and we suggest more research is important to better understand variations.

Although evidence on body composition in paediatrics is very rare given that it is a period of rapid growth and physical development [26, 27], available evidence suggests that the influence of stunting and its respective increased likelihood may be related to body composition, specifically muscle mass that is mainly located in the limbs and “the muscle arm area or circumference can be considered as a proxy estimate of muscle mass” and “muscle mass indices derived from mid-upper-arm-circumference are related to height-for-age” [27, 28]. A study by Myatt et al. (2009) also highlighted difference in body shape [body composition] using cross-sectional data of Ethiopian children, grouped by settled agrarian or semi-nomadic pastoralist, found that WHZ and MUAC observed similar prevalence estimates in agrarian children, but different estimates in pastoralist children with WHZ returning a significantly higher prevalence estimate compared to MUAC [29]. In summary, there is no single reason that explains the discrepancies found between WHZ and MUAC, which this analysis corroborates. This highlights the need to measure both indices at surveys and to use both figures for planning, as which is higher or lower varies from place to place and is very context specific. As for age and sex, it is still uncertain if the observed differences are due to the effect of direct influence of body composition or not, however, based on our findings we suggest assessing whether adding age and sex adjustments to MUAC improves performance. A similar recommendation has also been suggested by Laillou et al., (2014) using data from Cambodia and data from Ethiopia [30].

Lastly, our study shows that the observed discrepancy between WHZ and MUAC has programmatic implications for the treatment of acute malnutrition. In Mozambique many surveys estimate prevalence of acute malnutrition using WHZ and oedema only, despite collecting MUAC data. This means that programmes are planned and costed based on estimates derived from WHZ and oedema only. As shown in our results, using only WHZ and/oedema prevalence estimates accounts for 43.3% less than the actual number of children in need; using only MUAC and/oedema prevalence accounts for 32.1% less than the actual number of children in need (Fig. 6). For SAM, WHZ and/oedema-only represented 74.7% less children and SAM by MUAC and/oedema-only represented 8.4% less (Fig. 7) than the actual number of children in need. This means that using either GAM and SAM by WHZ and/oedema-only or GAM and SAM by MUAC and/oedema-only underestimates the number of children in need of treatment leading to unrealistic planning and costing figures affecting the entire value chain of the nutrition programme. This includes advocacy, forecast of nutrition supplies, staffing needs, fundraising and others, both for emergency and non-emergency programmes. This clearly shows that neither diagnostic criteria should be considered alone. Rather, a combination, including bilateral oedema, should be used through the combined prevalence estimates, to ensure accurate programme planning. Our results are consistent with findings reported by Humphreys et al. (2019) [31] in the context of Afghanistan where the Ministry of Public Health acknowledged the differences and adopted the use of the combined prevalence estimates towards more accurate programming [32]. Similarly, consistent with findings reported by Guesdon et., al (2020) [33], our study shows that use of one diagnostic criteria for admission to treatment programmes will exclude children in need of treatment (Fig. 2); we suggest that more analysis is necessary to determine the most effective way to identify children in need of treatment.

Mozambique has not yet adopted the use of combined prevalence estimates when calculating the number of children in need of treatment. Our analysis suggests therefore that the planning currently used is sub-optimal given that children in need are excluded by using either one method or the other. We therefore urge the Ministry of Health, Nutrition Cluster and other stakeholders to endorse the use of cGAM (cSAM and cMAM), first by promoting reporting of cGAM in population-based anthropometrics surveys and also when estimating number of children in need of treatment, both in emergency and non-emergency programmes, in order to more accurately reflect the number in need for advocacy, fundraising, procurement of nutrition supplies and other planning considerations. We suggest that further analysis is needed to analyse policy and programmatic implications for Mozambique, including the additional burden that needs to be reached, where that burden is located, if services are available and how services should be targeted. Further, since MUAC is the most simple and practical method to screen children at community level [34] further analysis should be carried out to assess if the power of MUAC can be increased to capture children that are diagnosed with wasting by WHZ (but not MUAC). This analysis should include severe (as well as global) wasting with adjustments for age and sex considered.

Despite using data from Mozambique only, given the fact that WHZ and MUAC diagnose different children as wasted, as previously documented in many countries including in Africa and Asia (Bilukha & Leidman, 2018 [32]; Grellety & Golden, 2016; Laillou et al., 2014; Wieringa et al., 2018 and others), we are confident that our findings are applicable to other settings, however the direction and extent of discordance would need to be contextualized. In other words, our findings around the programmatic implications caused by widely different caseload estimates between WHZ and/oedema only, MUAC and/oedema only and the combined prevalence estimates is also likely to be found in other countries and should be investigated further by country to ensure accurate advocacy and comprehensive response planning.

### Limitations

Some limitations of our study are described as follows: since we used secondary surveys conducted between 2017 and 2019, prevalence estimates presented in our study do not reflect the current situation in the respective areas. Also, even though our data came from 9 provinces (of 11) and all the three regions are represented in the sample, the study is not nationally representative as it covers only 28% of the 161 districts of Mozambique. The fact that we used SMART flags, which consider only children whose measurements are statistically plausible, means that some children with biologically plausible measurements may have been excluded from the study. In our regression models, besides age, sex and stunting, the database used did not have other factors of importance to model behaviour in relation to WHZ and MUAC diagnosis.

## Conclusions

In Mozambique, WHZ and MUAC rarely agree in their diagnostic classification of wasting in the same child, although positive and fair correlation between them exists. Age, sex and stunting all play a significant role on the influence of a wasting diagnosis classification using MUAC. Our study urges stakeholders to officially adopt the use of the combined prevalence estimates of acute malnutrition when calculating the number of children in need, both in routine and emergency programmes and this should be done by first promoting the inclusion of weight, height as well as MUAC and oedema measurements in all population based surveys that include anthropometry for children aged between 6 and 59 months. Additional analysis is recommended to assess the programmatic implications for Mozambique (such as targeting, financial, staffing and supply implications). Further analysis is also needed to ascertain the optimal level of MUAC to diagnose wasting at community or health facility level considering the discrepancies shown in this study.

## Data Availability

The datasets used and/or analysed during the current study are available from the corresponding author on reasonable request.

## Abbreviations

MUAC: Mid upper arm circumference
WHZ: Weight-for-height Z-score
GAM: Global acute malnutrition
cGAM: Combined prevalence of global acute malnutrition
SAM: Severe acute malnutrition
cSAM: Combined prevalence of severe acute malnutrition
cMAM: Combined prevalence of moderate acute malnutrition
SETSAN: Technical secretariat for food security and nutrition
ENA: Emergency nutrition assessment software
AOR: Associated odds ratio
mm: Millimetres
